# Kinematics and spatial structure analysis of TBM gunite robot based on D–H parameter method

**DOI:** 10.1038/s41598-024-64439-0

**Published:** 2024-06-13

**Authors:** Lianhui Jia, Shenyao Liu, Chenxu Cao, Yehao Kang, Ying Zhu, Lijun Wang, Donglai Xu, Ruixue Cheng

**Affiliations:** 1 China Railway Engineering Equipment Group Co, Zhengzhou, 450047 China; 2 School of Mechanical Engineering, North China University of Water Resources and Electric Power, Zhengzhou, 450045 China; 3 School of Computing, Engineering and Digital Technologies, Teesside University, Middlesbrough, TS1 3BX UK

**Keywords:** Electrical and electronic engineering, Mechanical engineering

## Abstract

In modern tunnel construction, TBM (Tunnel Boring Machine) plays a very important role. In response to the needs of tunnel wall reinforcement and TBM automated construction for tunnel construction, a shotcrete mechanism mounted on the TBM is designed. In order to evaluate the kinematic performance of the mechanism, this paper studies the forward and inverse kinematics and spatial architecture of the TBM shotcrete robot. Firstly, based on the D–H parameter method, the number of joints and links is determined and structural analysis is performed to obtain the robot's forward kinematics equation, achieving the mapping between joint space and pose space. Then, by determining the joint variables, the mapping of the end tool in Cartesian space is achieved. Finally, based on the Monte Carlo random sampling method, the workspace of the robot is constructed, and its reachability and flexibility within the robot workspace are evaluated. The performance of the device is verified by building a prototype, which meets the requirements well. Through the research in this paper, it can provide theoretical basis and guidance for the design and control of the shotcrete robot.

## Introduction

The construction of tunnel engineering is conducive to optimizing urban spatial structure, reducing travel time, improving transportation efficiency, and enhancing connectivity between regions^1^. Traditional tunnel construction involves drilling holes on the rock surface of the tunnel and filling them with explosives for blasting. However, tunnel construction is quite complex and this method is difficult to deal with various challenging situations such as quicksand, expansive geological formations, karst caves, and large amounts of water influx^2^. With the development of shield tunneling technology, the emergence of Tunnel Boring Machines (TBM) has completely replaced the traditional drilling and blasting method. Compared to traditional construction methods, TBM can achieve precise construction of the design tunnel axis (DTA)^3^, playing a crucial role in urban underground engineering construction, including metro systems, tunnels, and underground storage facilities. This represents a major breakthrough in the history of human engineering^4^.

During TBM construction, the tunneling speed is determined by controlling the penetration speed and the rotation speed of the cutterhead, but as the tunneling work progresses, it can lead to instability in the mechanical structure of the excavated arched surface^5^. To ensure the stability of the surrounding rock after lining the tunnel and the safety of the tunnel, it is often necessary to use steel mesh and shotcrete to form a coating that fits the tunnel surface, giving it resistance and shear forces to ensure stable stress on the tunnel, thus supporting the surrounding rock^6^. Further enhancing the strength of the surrounding rock can effectively prevent it from loosening or falling off. However, due to the uneven coating of the sprayed concrete and the foundation surface, it is difficult to ensure the construction quality. The shotcrete machinery still have problems in adapting to complex engineering environments and improving the durability of engineering structures^7^.

Currently, many scholars are trying to integrate machine learning, machine vision, and TBM to enhance the performance of the equipment^8,9^. Yun jiang Li and his team designed a PJR-D spraying robot, which controls the precise movement of the spray gun through a hydraulic manipulator. The structure is reasonable and the action is flexible^10^. KUKA AG also designed a spraying robot that can accurately control the spraying volume and position of the coating to ensure the uniformity and quality of the coating^11^. MEYCO (Norway) designed and manufactured a wet spraying trolley, which uses a wheeled chassis design and can move freely on the construction site, with climbing ability and mobility^12^. It can achieve efficient and continuous spraying during the construction process, and produce high-quality concrete spraying layers. Although many scholars have conducted research on shotcrete robots, there are still some problems. For example, the equipment is not intelligent enough, and manual adjustment of the nozzle angle and spraying speed is required through visual inspection using a remote control^13^. In addition, this method has problems such as large rebound, harsh construction environment, and high labor cost. The research in this article aims to solve this series of problems^14^.

The limited working environment of tunnel construction cannot accommodate large mechanical structures^15,16^. The trajectory of the structural end of many shotcrete machines cannot cover the working surface, and the tunnel construction environment is harsh, making existing machines unable to adapt to the harsh working environment. In response to the above practical problems, this article proposes a shotcrete robot design scheme, establishes a kinematic model for its working arm, and conducts trajectory planning to provide conditions for intelligent shotcrete.

Kinematics analysis aims to achieve the mapping of the robot from joint space to pose space, and then obtain its workspace^17^. Currently, there are many methods for kinematic modeling, including space vector polygon method, quaternion method, homogeneous coordinate representation method, D–H parameter method, and screw theory^18^. And there has been significant progress in recent years in this area of research^19^. Among them, describing the kinematics of the robot arm using screw theory has the advantages of beautiful form and clear physical meaning. However, this theory is relatively complex, requiring complex operations such as matrix operations and vector cross products in the specific calculation process, which poses problems of numerical stability and may introduce errors, resulting in inaccurate results^20^. The quaternion method has high complexity in understanding, and the concept and operation rules of quaternion are relatively abstract and complex^21^. There is a conversion overhead between quaternion and other forms of rotation expression (such as Euler angle and rotation matrix), which may increase the complexity and computational load of the calculation^22^. Although quaternion can save storage space compared to rotation matrix, it requires more storage space than Euler angle. In contrast, D–H parameter method is intuitive and simple to describe^23,24^.

The novelty of this article lies in the fact that the device creatively integrates the intelligent spray robot onto the TBM, which can completely replace traditional manual control. The designed structure is reasonable, with good motion performance and stability, providing a reliable foundation for further engineering applications. Moreover, it can meet the harsh environment of tunnel construction and limited working conditions.

In this paper, the D–H parameter method is used to perform a forward kinematics analysis of the designed TBM intelligent spray robot to determine the kinematic relationship between each joint of the robot and establish the forward kinematics equation of the robot. In addition, we conducted an inverse kinematics analysis and solved the inverse kinematics equation of the robot through analytical methods to calculate the motion parameters of each joint of the robot. This will help to control the motion state of the robot according to the required position and attitude of the end effector, and achieve intelligent spraying operations. Furthermore we solved the workspace of the robot to determine the effective working area that can be covered by the robot, and built a prototype to verify it, providing strong support for the planning and control of spraying tasks. Finally, the paper discusses the engineering application scenarios of the robot and where the advantages lie. To sum up, through structural analysis, forward and inverse kinematics analysis, and workspace solution, it can provide necessary foundation for intelligent operation and control of TBM intelligent spray robot.

## Spraying robot structure

### Robot structure design

**Figure 1:**
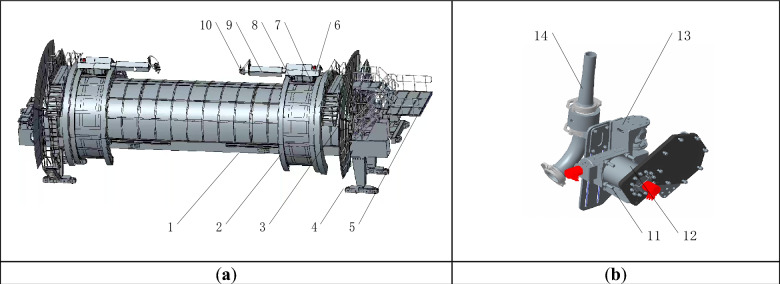
Schematic diagram of the spraying robot structure. (**a**) Schematic diagram of the overall structure of the spraying robot. (**b**) Schematic diagram of the spray head structure of the spraying robot. In the figure, 1—The gunite part of the TBM; 2—Rack; 3—Baffle plate; 4—Guide pulley; 5—Guide pulley; 6—Cylinder protection cover; 7—Driving motor; 8—Oil cylinder; 9—Mechanical arm protective cover; 10—Nozzle part; 11—Eccentric; 12—Oscillating cylinder; 13—Double acting motor; 14—Sprayer.

The overall structure of the shotcrete robot is designed for the needs of practical construction, and its three-dimensional structure is shown in Fig. 1.

In order to enable the shotcrete robot to be mounted on the TBM and complete the shotcrete work of the circular tunnel as the tunneling work progresses, the robot is equipped with guide rails and pulleys below. The rotary motion of the robot arm is achieved through a gear and rack structure, and the driving wheel drives the motor located below the robot arm. In order to protect the meshing part of the robot arm, a protective cover is installed at the meshing part. At the same time, in order to achieve longitudinal movement of the robot arm in the working area, a hydraulic cylinder is added, and the cylinder is placed below the robot arm.

The end of the robot arm is equipped with a nozzle, a double-acting motor, two swinging oil cylinders, and an encoder at the end of the oil cylinder. During operation, the encoder receives commands to control the rotation angle, which in turn controls the working angle of the nozzle. With the double-acting motor connected to an eccentric wheel, the nozzle can make a circular motion as the eccentric wheel rotates, allowing for a wider spraying range.

### Robot operation parameters

**Figure 2:**
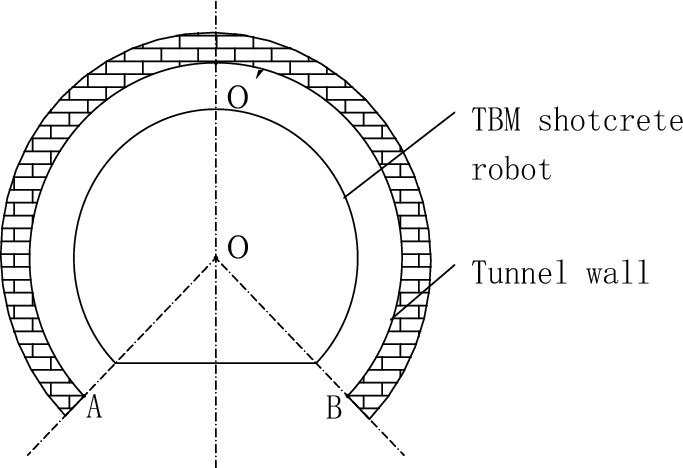
Robot working range.

Some parameters of the mechanism are shown in Table 1. The shotcrete robot is equipped with an open-type TBM with a diameter of 6400 mm, and the tunnel excavation radius is 3200 mm. The machine can move horizontally along the tracks A and B. The AB area, i.e. the bottom of the tunnel where the up-arches are laid, has a 270° rotating circumference, and the turnaround movement is realized by gears engaging with the large gear ring of the basic frame. Due to structural constraints and the fact that no grouting is required for the up-arch, the machine's working range is mainly in the AO′B area of the tunnel wall, where ∠AOB = 270°. The spraying range is shown in Fig. 2.

**Table 1:** Basic parameters of robots.

Parameter	Numerical value
Freedom	5
Scope of work	1.5π
Gunite quantity	20 m^3^/h
End positioning accuracy	± 15 mm
End movement speed	0.10 m/s
Manipulator speed	0.05 m/s

### Coordinate system of robotic arm

Due to the arched working environment of the shotcrete robot, the working space has high requirements on the structure of the robot arm, and it must be considered whether the trajectory of its end effector will overlap with the working space. The manipulator designed in this article meets the general calculation method for robot arms, and can further determine whether the parameters of the robot arm meet this condition through kinematic calculations.

The kinematics of the manipulator arm is the study of the geometric relationship of arm motion. The robot manipulator can be seen as an open kinematic chain, which is composed of a series of links connected by rotating or moving joints in series. One end of the open chain is fixed to the base, and the other end is free, with a terminal effector mounted to complete the work.

In order to study the relationship between the links of the manipulator, a coordinate system is fixed at the joint and the relationship between the coordinate systems is described. Denavit and Hartenberg proposed a general method using homogeneous transformations to describe the spatial geometric relationship of each link relative to a fixed reference frame. A 4 × 4 homogeneous transformation matrix is used to describe the spatial relationship between two adjacent links, thereby deriving an equivalent homogeneous transformation matrix for the end effector relative to the reference frame and establishing the motion equation of the manipulator.

**Figure 3:**
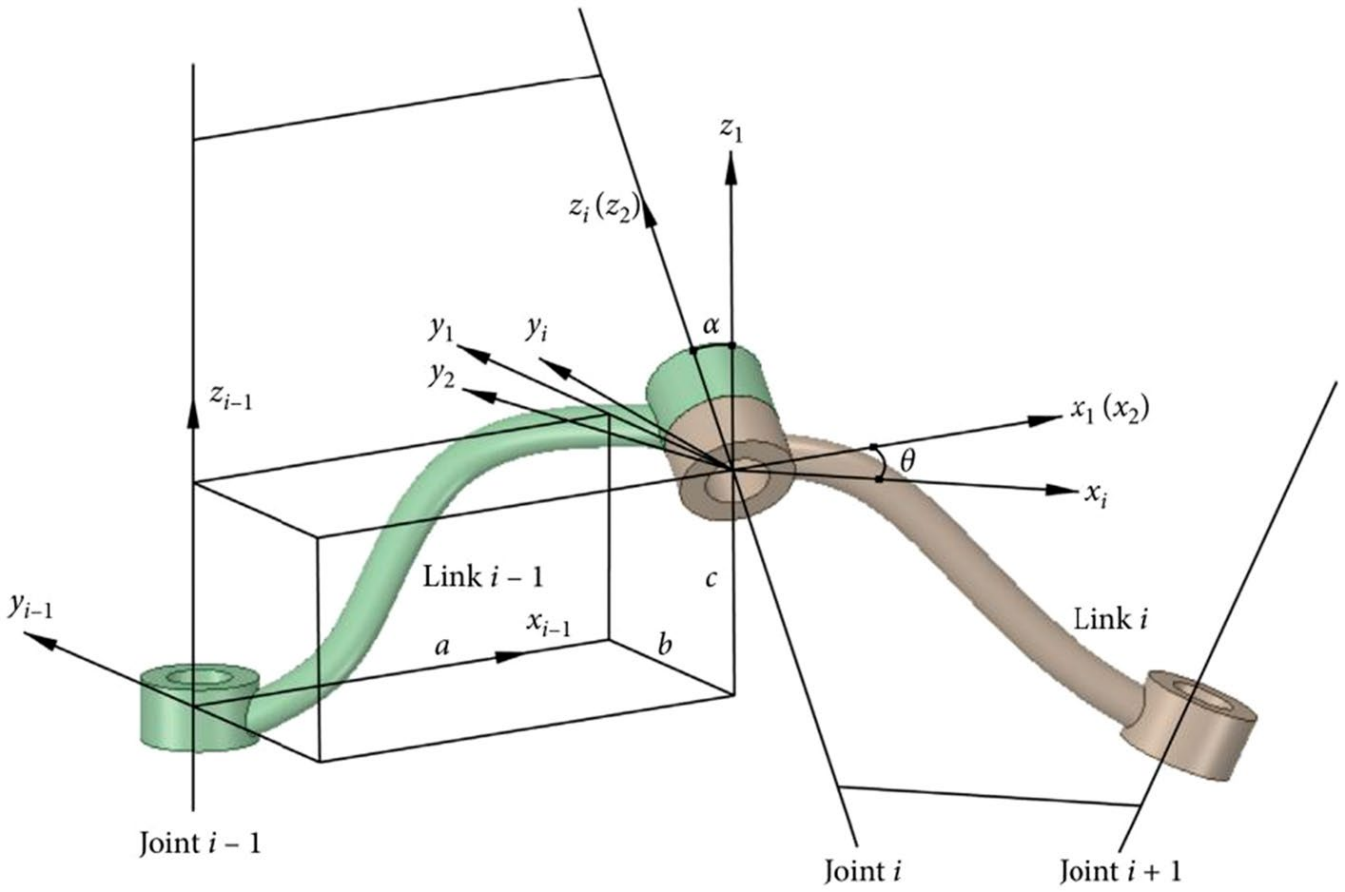
Modeling method when adjacent joints are not parallel^25^.

Take the modeling method when adjacent links are not parallel as an example, as shown in Fig. 3. First, number the joints and links to the end effector as the end, and number the reference as 0. Numbering the joints sequentially from 1 to n, joint i connects link i-1 and link i. Secondly, in the Cartesian coordinate system, the Z axis coincides with the joint axis, the X axis is parallel to the common normal line of two adjacent joint axes, and the Y axis is determined by the Cartesian right-hand rule. The joint coordinate system i is embedded in the link i. Finally, when the first joint variable is 0, it is specified that 0 coincides with 1. For the end coordinate system n, place the origin of the coordinate system at the desired position on the joint axis. The X axis direction can be arbitrarily selected, but it is always desirable to choose a coordinate system n such that the link parameter is 0.

## Kinematic analysis

In the field of industrial robot control, the space describing the motion state and posture of robots is usually divided into pose space, joint space, drive space, and detection space. In this article, the pose space is mainly used to describe the position and posture of the spraying robot in Cartesian coordinates; the joint space is composed of joint angles of each joint, which is a 4 × 1 joint vector. The drive space is composed of the variation of the drive mechanism, such as the rotation angle of the drive motor meshing with the large gear ring, the extension of the first telescopic joint cylinder, the rotation angle of the wrist swing cylinder, and the rotation angle of the end swing cylinder.

In robot control algorithms, the forward and inverse kinematics problem is a classic transformation problem involving calculations between the pose space and joint space. Specifically, the forward kinematics problem refers to how to calculate the position and orientation of the robot's end effector in Cartesian coordinates given the joint angles of the robot. Conversely, the inverse kinematics problem refers to how to calculate the joint angles of each joint given the position and orientation of the robot's end effector in Cartesian coordinates.

**Figure 4:**
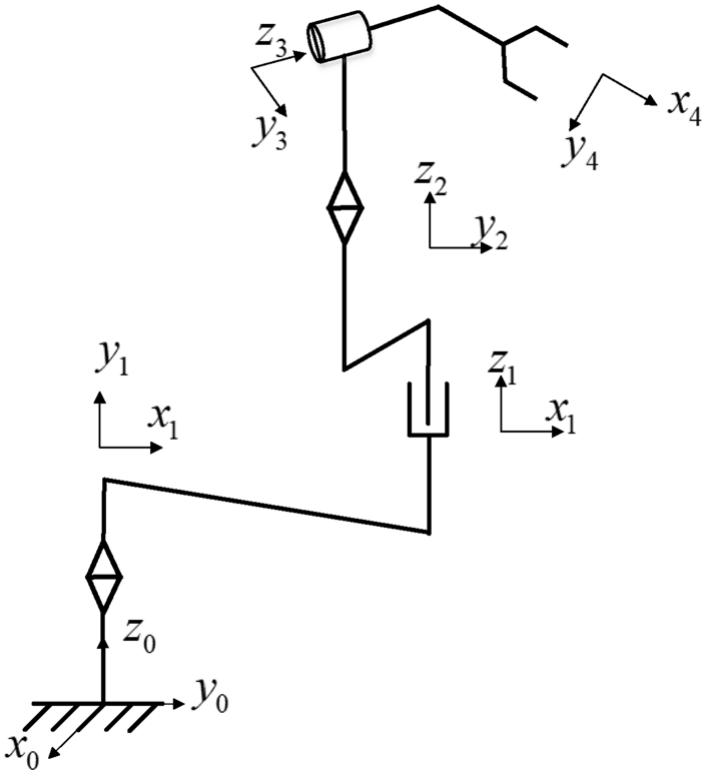
The coordinate system of the spraying robot.

Using the Denavit–Hartenberg formalism, the coordinates of the links and joints of the robot arm are constructed, and then the end of the link is taken as the coordinate origin, as shown in Fig. 4. The D–H parameter table is also obtained, as shown in Table 2.

**Table 2:** DH parameter.

Link	Corner	Offset	Link length	Link rod angle
1	$\theta_{1}$	*k*_1_	*k*_2_	0
2	$$- {\raise0.7ex\hbox{$\pi $} \!\mathord{\left/ {\vphantom {\pi 2}}\right.\kern-0pt} \!\lower0.7ex\hbox{$2$}}$$	*d*_2_	*k*_3_	0
3	$$\theta_{3}$$	*k*_4_	0	$$- {\raise0.7ex\hbox{$\pi $} \!\mathord{\left/ {\vphantom {\pi 2}}\right.\kern-0pt} \!\lower0.7ex\hbox{$2$}}$$
4	$\theta_{4}$	*k*_5_	*k*_6_	0

As shown in the Fig. 4, the spraying robot has four active components, including one high pair and three low pairs. The number of degrees of freedom of the shotcrete robot:

1 $$F = 3n - (P_{h} + 2P_{l} )$$

In the calculation formula of degrees of freedom, where the *F* is the Number of free members, *n* is the Number of free members, *P*_*h*_ is the Higher accessory number, *P*_*l*_ is the Lower accessory number. Substitute *n* = 4, *P*_h_ = 1, *P*_l_ = 3 into the formula and calculate the degree of freedom as 5.

### Positive kinematics analysis

According to the D–H parameter table, the parameters of each joint are obtained, especially the length, displacement, phase, and rotation angle of the axis. These parameters are used to draw a matrix equation (which relates the angles in the joint to the desired coordinates of the working tool), represented by the homogeneous transformation matrix ${}^{i - 1}T_{i}$, to represent the pose transformation relationship between the coordinate system of link i and the coordinate system of link *i *− 1:

2 $${}^{i - 1}T_{i} = {\text{Rot}}(z,\;\theta_{i} ){\text{Trans}}(0,\;0,\;d_{i} ){\text{Trans}}(a_{i} ,\;0,\;0){\text{Rot}}(x,\;\alpha_{i} )$$

After unfolding the above equation, it can be concluded that:

3 $${}^{i - 1}T_{i} = \left[ {\begin{array}{*{20}c} {\cos \theta_{i} } & {\quad - \cos \alpha_{i} \sin \theta_{i} } & {\quad \sin \alpha_{i} \sin \theta_{i} } & {\quad a_{i} \cos \theta_{i} } \\ {\sin \theta_{i} } & {\quad \cos \alpha_{i} \cos \theta_{i} } & {\quad - \sin \alpha_{i} \cos \theta_{i} } & {\quad a_{i} \sin \theta_{i} } \\ 0 & {\quad \sin \alpha_{i} } & {\quad \cos \alpha_{i} } & {\quad d_{i} } \\ 0 & {\quad 0} & {\quad 0} & {\quad 1} \\ \end{array} } \right]$$

Substituting the linkages and key parameters in Table 2 into the above formula, four link transformation matrices can be obtained:

4 $${}^{0}T_{1} = \left[ {\begin{array}{*{20}c} {\cos \theta_{1} } & {\quad - \sin \theta_{1} } & {\quad 0} & {\quad k_{1} \cos \theta_{1} } \\ {\sin \theta_{1} } & {\quad \cos \theta_{1} } & {\quad 0} & {\quad k_{1} \sin \theta_{1} } \\ 0 & {\quad 0} & {\quad 1} & {\quad k_{2} } \\ 0 & {\quad 0} & {\quad 0} & {\quad 1} \\ \end{array} } \right]$$

5 $${}^{1}T_{2} = \left[ {\begin{array}{*{20}c} 0 & {\quad 1} & {\quad 0} & {\quad 0} \\ { - 1} & {\quad 0} & {\quad 0} & {\quad - k_{3} } \\ 0 & {\quad 0} & {\quad 1} & {\quad d_{2} } \\ 0 & {\quad 0} & {\quad 0} & {\quad 1} \\ \end{array} } \right]$$

6 $${}^{2}T_{3} = \left[ {\begin{array}{*{20}c} {\cos \theta_{3} } & {\quad 0} & {\quad \sin \theta_{3} } & {\quad 0} \\ {\sin \theta_{3} } & {\quad 0} & {\quad \cos \theta_{3} } & {\quad 0} \\ 0 & {\quad 1} & {\quad 0} & {\quad k_{4} } \\ 0 & {\quad 0} & {\quad 0} & {\quad 1} \\ \end{array} } \right]$$

7 $${}^{3}T_{4} = \left[ {\begin{array}{*{20}c} {\cos \theta_{4} } & {\quad - \sin \theta_{4} } & {\quad 0} & {\quad k_{6} \cos \theta_{4} } \\ {\sin \theta_{4} } & {\quad \cos \theta_{4} } & {\quad 0} & {\quad k_{6} \sin \theta_{4} } \\ 0 & {\quad 0} & {\quad 1} & {\quad k_{5} } \\ 0 & {\quad 0} & {\quad 0} & {\quad 1} \\ \end{array} } \right]$$

Therefore, the pose of the end nozzle of the intelligent spraying robot in the base coordinate system can be expressed as:

8 $${}^{0}T_{4} = {}^{0}T_{1} {}^{1}T_{2} {}^{2}T_{3} {}^{3}T_{4} = \left[ {\begin{array}{*{20}c} {n_{x} } & {\quad o_{x} } & {\quad a_{x} } & {\quad p_{x} } \\ {n_{y} } & {\quad o_{y} } & {\quad a_{y} } & {\quad p_{y} } \\ {n_{z} } & {\quad o_{z} } & {\quad a_{z} } & {\quad p_{z} } \\ 0 & {\quad 0} & {\quad 0} & {\quad 1} \\ \end{array} } \right]$$

After expansion, the transformation matrix from the base coordinate system to coordinate system 4 for the spraying robot can be obtained:

9 $$^{0} T_{4} = \left[ {\begin{array}{*{20}c} {c_{4} (c_{3} s_{1} + s_{3} c_{1} )} & {\quad - s_{4} (c_{3} s_{1} + s_{3} c_{1} )} & {\quad c_{3} (c_{1} - s_{3} s_{1} )} & {\quad k_{5} (c_{3} c_{1} - s_{3} s_{1} ) + k_{2} c_{1} + k_{3} s_{1} + k_{6} c_{4} (c_{3} s_{1} + s_{3} c_{1} )} \\ { - c_{4} (c_{3} c_{1} - s_{3} s_{1} )} & {\quad s_{4} (c_{3} c_{1} - s_{3} s_{1} )} & {\quad c_{3} s_{1} + s_{3} c_{1} } & {\quad k_{5} (c_{3} s_{1} + s_{3} c_{1} )k_{3} c_{1} + k_{2} s_{1} - k_{6} c_{4} (c_{3} c_{1} - s_{3} s_{1} )} \\ { - s_{4} } & {\quad - c_{4} } & {\quad 0} & {\quad k_{1} + d_{2} + k_{4} - k_{6} s_{4} } \\ 0 & {\quad 0} & {\quad 0} & {\quad 1} \\ \end{array} } \right]$$

For simplicity, use* Cn* instead of $\cos \theta {\text{n}}$, *Sn* instead of $\sin \theta n$.

### Reverse kinematics analysis

The purpose of inverse kinematics analysis is to convert the pose of the end effector in Cartesian coordinates into variables for each joint, which directly involves motion analysis and trajectory planning. In order for the robot to reach a specified position coordinate, it is necessary to solve the problem of how to make each joint move, which is known as the inverse kinematics problem. Inverse kinematics is generally used in motion analysis, trajectory planning, and other fields, and is the basic motion control technology for intelligent operations of the robot. Compared to forward kinematics, inverse kinematics research is more complex and has greater practical significance.

There are two common methods for inverse kinematics of robotic arms: analytical method and numerical method. The analytical method obtains analytical solutions by solving the inverse kinematics equations of the robotic arm. The numerical method obtains approximate solutions through iterative approximation. Compared to the numerical method, the analytical method has better real-time performance and higher accuracy, but it cannot be applied to solve inverse kinematics for all configurations of robotic arms. The advantage of the numerical method is its greater versatility.

This article uses analytical methods to solve the inverse kinematics problem. The steps for solving each joint variable are as follows:

**(1) Solution of** $$\theta 4$$.

Firstly, the position and orientation matrix of the TBM robot end nozzle motion path point calculated in Cartesian coordinates is shown below.

10 $$\left[ {\begin{array}{*{20}c} {n_{x} } & {\quad o_{x} } & {\quad a_{x} } & {\quad p_{x} } \\ {n_{y} } & {\quad o_{y} } & {\quad a_{y} } & {\quad p_{y} } \\ {n_{z} } & {\quad o_{z} } & {\quad a_{z} } & {\quad p_{z} } \\ 0 & {\quad 0} & {\quad 0} & {\quad 1} \\ \end{array} } \right] = {}^{0}T_{1} {}^{1}T_{2} {}^{2}T_{3} {}^{3}T_{4}$$

Left multiply the left and right sides of Eq. (10) by the inverse matrix ${}^{0}T_{1}^{ - 1}$ of ${}^{0}T_{1}$ to obtain:

11 $${}^{0}T^{ - 1} {}^{0}T_{4} = {}^{1}T_{2}^{2} T_{3}^{3} T_{4}$$

Record $T_{234L} = {}^{0}T_{1}^{ - 1} {}^{0}T_{4}$, $T_{234R} = {}^{1}T_{2} {}^{2}T_{3} {}^{3}T_{4}$ is expanded as:

12 $$T_{234L} = \left[ {\begin{array}{*{20}c} {n_{x} c_{1} + n_{y} s_{1} } & {\quad o_{x} c_{1} + o_{y} s_{1} } & {\quad a_{x} c_{1} + a_{y} s_{1} } & {\quad p_{x} c_{1 } - k_{2} + p_{y} s_{1} } \\ {n_{y} c_{1} - n_{x} s_{1} } & {\quad o_{y} c_{1} - o_{x} s_{1} } & {\quad a_{y} c_{1} - a_{x} s_{1} } & {\quad p_{y} c_{1} - p_{x} s_{1} } \\ {n_{z} } & {\quad o_{z} } & {\quad a_{z} } & {\quad p_{z} - k_{1} } \\ 0 & {\quad 0} & {\quad 0} & {\quad 1} \\ \end{array} } \right]$$

13 $$T_{234R} = \left[ {\begin{array}{*{20}c} {c_{4} s_{3} } & {\quad - s_{4} s_{3} } & {\quad c_{3} } & {\quad k_{5} c_{3} + \, k_{6} c_{4} s_{3} } \\ { - c_{4} c_{3} } & {\quad s_{4} c_{3} } & {\quad s_{3} } & {\quad k_{5} s_{3} - k_{3} - k_{6} c_{4} c_{3} } \\ { - s_{4} } & {\quad - c_{4} } & {\quad 0} & {\quad d_{2} + \, k_{4} - k_{6} s_{4} } \\ 0 & {\quad 0} & {\quad 0} & {\quad 1} \\ \end{array} } \right]$$

According to the matrix property, the corresponding elements are equal, and the element in the first column of the third row is equal to the element in the second column of the third row, which leads to:

14 $$\left\{ {\begin{array}{*{20}c} {n_{z} = - s_{4} } \\ {o_{z} = - c_{4} } \\ \end{array} } \right.$$

So, $$\theta_{4} = a\tan 2(n_{z} ,o_{z} )$$.

**(2) Solution of** ***d***_**2**_.

Similarly, based on the equality of corresponding elements in the matrix, that is, the equality of elements in the third row and fourth column, we obtain:

15 $$d_{2} = p_{z} - k_{1} - k_{4} + k_{6} s_{4}$$

**(3) Solution of** $$\theta 1$$.

Left multiply the left and right sides of (10) by the inverse matrix ${}^{0}T_{1}^{ - 1}$ of ${}^{0}T_{1}$, multiply the inverse matrix ${}^{3}T_{4}^{ - 1}$ of ${}^{3}T_{4}$ on the right:

16 $${}^{0}T_{1}^{ - 1} {}^{0}T_{4} {}^{3}T_{4}^{ - 1} = {}^{1}T_{2} {}^{2}T_{3}$$

Record $T_{23L} = {}^{0}T_{1}^{ - 1} {}^{0}T_{4}^{3} T_{4}^{ - 1}$, $T_{23R} = {}^{1}T_{2} {}^{2}T_{3}$ is expanded as:

17 $$T_{23L}^{T} = \left[ {\begin{array}{*{20}c} {c_{4} (n_{x} c_{1} + n_{y} s_{1} ) - s_{4} (o_{x} c_{1} + o_{y} s_{1} )} & {\quad c_{4} (n_{y} c_{1} - n_{x} s_{1} ) - s_{4} (o_{y} c_{1} - o_{x} s_{1} )} & {\quad n_{z} c_{4} - o_{z} s_{4} } & {\quad 0} \\ {s_{4} (n_{x} c_{1} + n_{y} s_{1} ) + c_{4} (o_{x} c_{1} + o_{y} s_{1} )} & {\quad s_{4} (n_{y} c_{1} - n_{x} s_{1} ) + c_{4} (o_{y} c_{1} - o_{x} s_{1} )} & {\quad o_{z} c_{4} + n_{z} s_{4} } & {\quad 0} \\ {a_{x} c_{1} + a_{y} s_{1} } & {\quad a_{y} c_{1} - a_{x} s_{1} } & {\quad a_{z} } & {\quad 0} \\ {p_{x} c_{1} - k_{5} (a_{x} c_{1} + a_{y} s_{1} ) - k_{6} (n_{x} c_{1} + n_{y} s_{1} ) - k_{2} + p_{y} s_{1} } & {\quad p_{y} c_{1} - k_{6} (n_{y} c_{1} - n_{x} s_{1} ) - k_{5} (a_{y} c_{1} - a_{x} s_{1} ) - p_{x} s_{1} } & {\quad p_{z} - k_{1} - a_{z} k_{5} - k_{6} n_{z} } & {\quad 1} \\ \end{array} } \right]$$

18 $$T^{T}_{23R} = \left[ {\begin{array}{*{20}c} { \, s_{3} } & {\quad - c_{3} } & {\quad 0} & {\quad 0} \\ 0 & {\quad 0} & {\quad - 1} & {\quad 0} \\ {c_{3} } & {\quad s_{3} } & {\quad 0} & {\quad 0} \\ 0 & {\quad - k_{3} } & {\quad d_{2} + k_{4} } & {\quad 1} \\ \end{array} } \right]$$

According to the equality of corresponding elements in the matrix, that is, the equality of the element in the first column of the second row and the element in the second column of the second row, we obtain:

19 $$\left\{ {\begin{array}{*{20}c} {s_{4} (n_{x} c_{1} + n_{y} s_{1} ) + c_{4} (o_{x} c_{1} + o_{y} s_{1} ) = 0} \\ {s_{4} (n_{y} c_{1} - n_{x} s_{1} ) + c_{4} (o_{y} c_{1} - o_{x} s_{1} ) = 0} \\ \end{array} } \right.$$

Adding the two equations and simplifying, we obtain:

20 $$\theta_{1} = \arctan 2\left( { - \left( {s_{4} n_{x} + c_{4} o_{x} + s_{4} n_{y} + c_{4} o_{y} } \right),\left( {s_{4} n_{y} + c_{4} o_{y} - s_{4} n_{x} - c_{4} o_{x} } \right)} \right)$$

**(4) Solution of** $$\theta$$_**3**_

According to the equality of corresponding elements in the matrix, that is, the equality of the first column element in the third row and the second column element in the third row, we obtain:

21 $$\left\{ \begin{gathered} axc1 + ays1 = c3 \hfill \\ ayc1 - axs1 = s3 \hfill \\ \end{gathered} \right.$$

So, $$\theta_{3} = a\tan 2(a_{y} c_{1} - a_{x} s_{1} ,a_{x} c_{1} + a_{y} s_{1} )$$.

So far, the inverse kinematics solutions of each joint of the shotcrete robot have been solved. In summary, multiple inverse solutions can be obtained for the TBM shotcrete robot to reach any position. In the actual control process, the optimal solution needs to be selected. The inverse solutions that exceed the joint range are deleted, and the inverse solutions that meet the joint range are subtracted from the joint variables of the previous pose. Following the principle of “moving small joints more and large joints less”, the difference is weighted, and the smallest set is selected as the optimal solution.

22 $${\text{min}}\sum\limits_{i = 1}^{6} {\left| {\omega_{i} \Delta_{i} } \right|}$$

where, $\omega_{i}$ is the weighting coefficient of joint variables; ${\Delta }_{i}$ is the joint variable.

## Workspace calculation and prototype verification

This article uses Robotics Toolbox to perform kinematic simulation of the TBM shotcrete robot to verify the correctness of the kinematic analysis, and draws the working space of the robot arm based on the Monte Carlo method. The virtual simulation results are in line with expectations. On this basis, we built a prototype. The motion trajectory and speed of the prototype under different working conditions meet the expected design requirements, indicating that the mechanical structure can effectively complete the established motion tasks.

### Workspace analysis

The workspace represents the geometric space that can be reached by the end effector of a TBM shotcrete robot, and is one of its performance indicators. Currently, the calculation methods of workspace mainly include analytical method, numerical method, graphical method, etc. Among them, the analytical method determines the boundary of the workspace by envelope curve, which is not intuitive enough. The graphical method is simple and intuitive, but when the robot has more degrees of freedom, the solution is more cumbersome. As a numerical calculation method based on random sampling, Monte Carlo method is widely used in solving robot workspace problems. Monte Carlo method can be used to solve various complex robot workspace problems, with fast calculation speed, easy to achieve graphical display, and it can estimate unknown functions or probability distributions through random sampling and statistical analysis, and provide approximate solutions. Therefore, it is commonly used.

The principle of using the Monte Carlo method to draw the workspace is as follows: first, based on the calculation results of the forward kinematics of the TBM grouting robot, obtain the position coordinate formula of its end nozzle. Second, randomly sample the joint values of each joint of the robot arm, and then substitute the randomly sampled results into the forward kinematics equation to calculate, obtain the position coordinate formula of its end nozzle. Finally, draw a point cloud set to obtain the workspace of the robot arm.

Among them, the joint parameters of the TBM shotcrete robot are *k*_1_ = 1266.3, *k*_2_ = 3964.5, *k*_3_ = 169.5, *k*_4_ = 545.1, *k*_5_ = 195, *k*_6_ = 688.8, the value range of* d*_2_ is 814–6814 mm.

If the workspace is W(P), its workspace can be described as:

23 $$W\left( P \right) = \left\{ {\begin{array}{*{20}c} {p_{x} \left( \theta \right)} \\ {p_{y} \left( \theta \right)} \\ {p_{z} \left( \theta \right)} \\ \end{array} } \right.,\quad \theta \in Q$$

**Figure 5:**
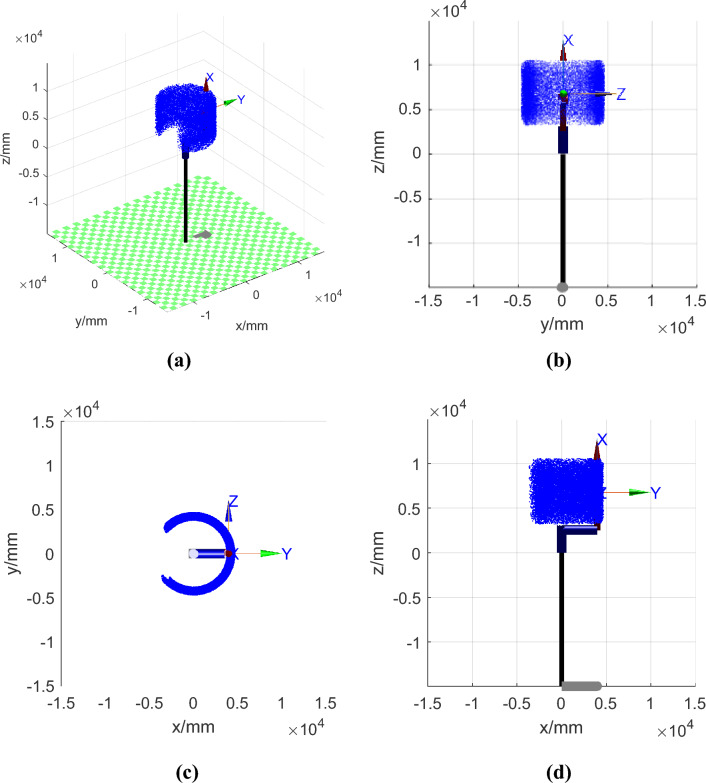
Workspace analysis. (**a**) Three-dimensional cloud map of the workspace. (**b**) Projection of the workspace on the yoz plane. (**c**) Projection of the workspace on the xoy plane. (**d**) Projection of the workspace on the xoz plane.

In the formula, *P*_x_, *P*_y_, *P*_Z_, respectively represent the coordinate values of the end spray head of the TBM intelligent spray robot in the base coordinate system; θ is the joint vector of the TBM intelligent spray robot;

Substituting the position and orientation Eq. (10) of the end nozzle of the intelligent shotcrete robot calculated by forward kinematics in the base coordinate system into the calculation, we obtain the three-dimensional cloud map of the robot arm. After conducting workspace analysis, we obtained the results shown in Fig. 5.

### Prototype construction and experimental verification

**Figure 6:**
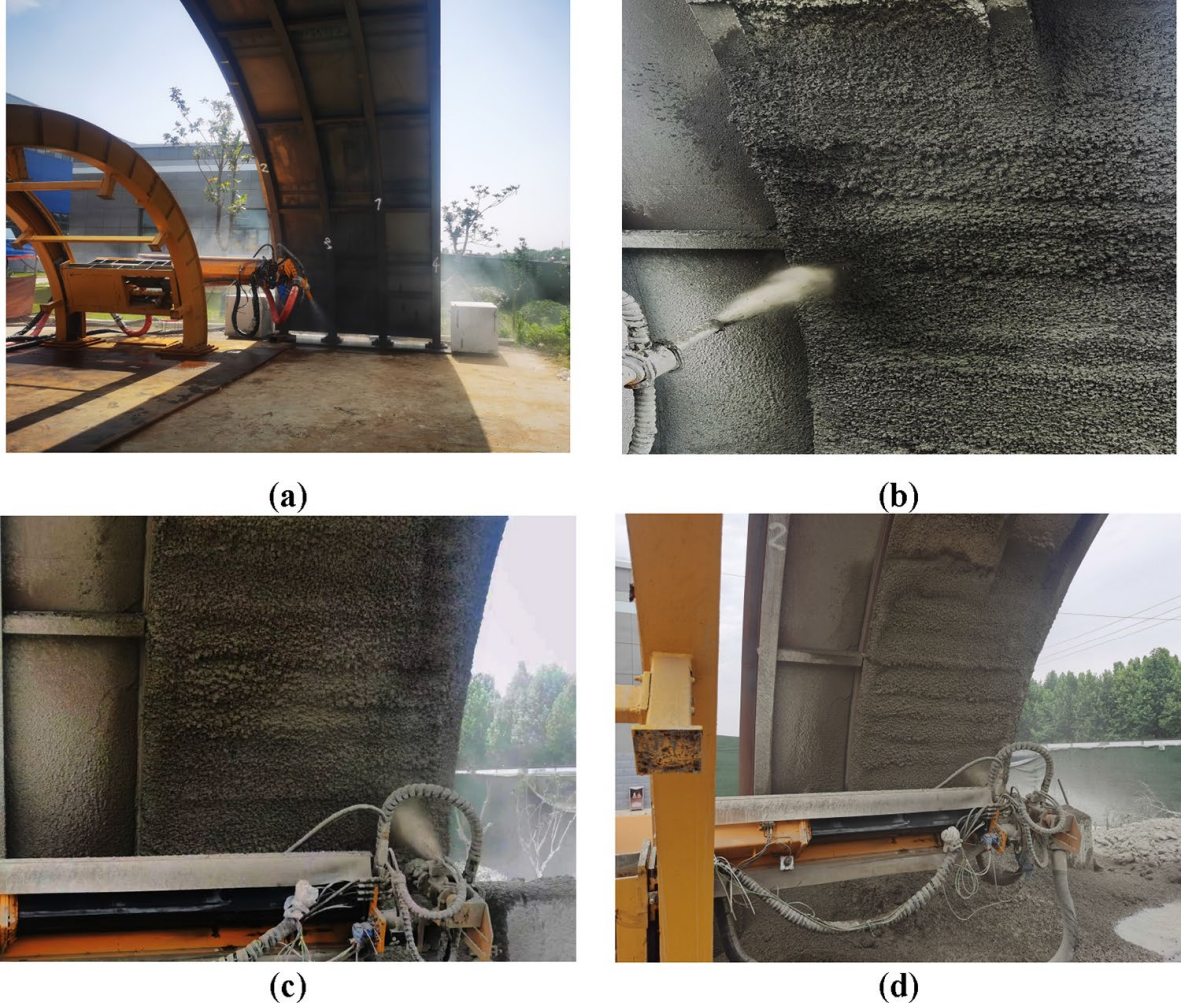
Experimental verification (**a**) robot structure, (**b**) first spraying experiment, (**c**) uniform spraying experiment, (**d**) uniform spraying experiment results.

Three-dimensional visualization simulation, prototype experiment, and field experiment are three main ways to verify the robot theory. Based on the three-dimensional model of the spraying robot, a spraying robot prototype is designed to control the prototype to complete the spraying operation to verify the correctness of the kinematics calculation results. At the same time, structural debugging is carried out, and automated spraying operation experiments under simulated conditions are carried out to verify the correctness and feasibility of the spraying robot structure proposed in this paper. The prototype structure is shown in Fig. 6a.

In the experiment, steel arches were used to simulate the tunnel environment for spraying experiments, and the spraying area was part of the arch. The specific spraying parameters were: the nozzle was swung left and right by 90°, the nozzle was swung up and down by 45°, and the nozzle was at an angle of about 90° to the tunnel surface; the concrete pumping flow rate was 10 m^3^/h, and the spraying process was repeated 5 times. The spraying process is shown in Fig. 6c, and the spraying results are shown in Fig. 6d.

In this experiment, the thickness of sprayed concrete after solidification was 50 mm, and it was examined by hammering method, and no air drum was found to exist, which satisfied the industry standard. The lining flatness index was 5%, and the industry standard requires 20% for this. After testing, the sprayed concrete layer can be initially solidified after one hour of spraying. At the same time, through the surface observation, did not find cracks leakage spraying as well as the phenomenon of shedding.

The experimental results demonstrate that the end of the robotic arm actuator successfully simulates tunnel space without encountering collisions. The working area encompasses the tunnel wall, ensuring effective spraying of slurry to meet the requirements of tunnel construction. No abnormal vibrations, noise, or failures were observed during the operation of the prototype. Furthermore, the trajectory of the nozzle exhibits minimal fluctuation when moving between different target points, indicating a high level of stability and reliability in the prototype.

## Discussion

The TBM intelligent slurry spraying machine described in this paper is able to realize unmanned, safe, accurate and efficient tunnel primary lining operation. According to the above analysis, it is expected to become a brand-new technical means for the initial lining of tunnel in TBM construction section, so it is combined with 10 m class TBM to comprehensively discuss the TBM intelligent slurry spraying robot mounting scheme, the slurry spraying process, and its efficiency is analyzed.

### TBM intelligent grouting system carrying solution

TBM is the construction tool in hard rock tunnel construction. According to the literature research and field construction experience, in the process of TBM tunnel excavation, the initial lining operation is usually done by manual remote control operation of TBM wet spraying machine to complete the initial lining operation on the excavated tunnel surface. However, the construction environment is harsh, and the construction quality and efficiency cannot be guaranteed. Therefore, the use of TBM intelligent slurry spraying robot instead of TBM wet spraying machine, can use radar, displacement, angle and other sensors, automatic identification of tunnel section characteristics, automatic calculation of TBM intelligent slurry spraying joints position, automatic execution and completion of slurry spraying initial lining action, to realize the unmanned, safe, accurate and efficient tunnel lining operation in the process of TBM construction.

### TBM intelligent slurry spraying process

**Figure 7:**
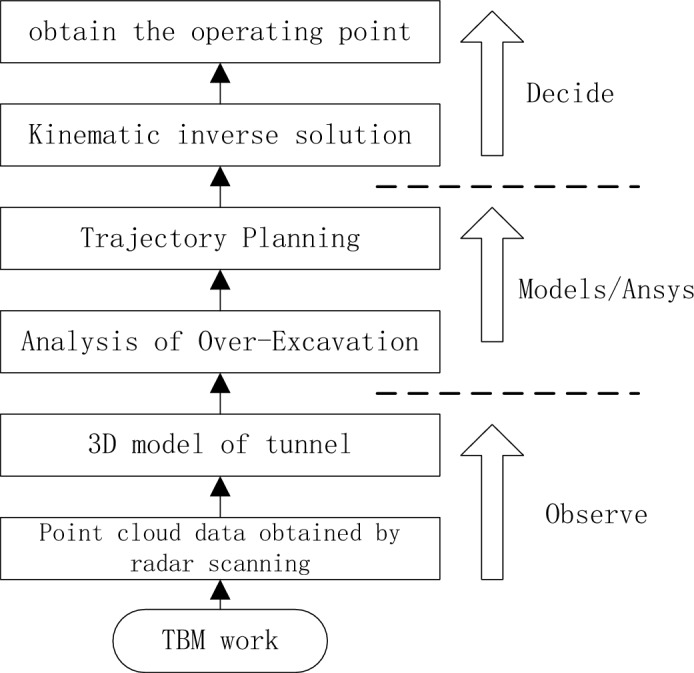
The slurry spraying process.

TBM intelligent grouting by radar scanning tunnel point cloud data, based on the data to realize the reconstruction of the three-dimensional model. At the same time, the calculation of the tunnel sub-area over and under excavation amount, through the path planning, kinematics inverse solution to convert into the slurry spraying mechanical arm of the joints of the amount of movement, the control system integration, and control of the slurry spraying mechanical arm to implement the slurry spraying operation until the tunnel surface to be sprayed to complete the initial lining operation. The slurry spraying process is shown in Fig. 7.

### TBM intelligent slurry robot primary lining efficiency analysis

Take 10 m class TBM as an example, the industry standard requirements, the initial lining thickness of 50 mm, a single class of initial lining interval of about 5 m, the total amount of concrete required about 3 m^3^, combined with the slurry comprehensive rebound rate of 15%, the actual amount of concrete required 3.5 m^3^. TBM intelligent slurry spraying system of 20 m^3^/h, combined with the time of wetting, scanning and other processes, the overall slurry spraying initial lining efficiency as shown in the table, compared with the traditional manual slurry spraying efficiency increased by 30%, the efficiency of the slurry spraying system is higher than that of the traditional manual spraying system. Compared with the traditional manual slurry spraying, the efficiency is improved by 30%. Detailed information is shown in Table 3.

**Table 3.** 10 m class TBM slurry spraying man-hour efficiency statistics table.

Working procedure	Single process time/min (TBM intelligent slurry robot)	Single process time/min (manual operation)
Pipe lubrication	30	30
Tunnel section scanning	0.5	–
Calculation of over and under excavation	0.5	–
Route planning	0.1	–
Slurry spraying action	40	70
Pipe flushing	15	20
Total	86.1	120

## Conclusion

In this paper, a new type of tunnel slurry spraying robot mechanism is designed based on Duffy-Harrington model for tunnel slurry spraying operation, which is aimed at the problems of low degree of intelligence of the slurry spraying robot, poor smoothness of the robot arm movement and large limitation of the workspace, etc. The mechanism is described by the actual TBM as the mounting object. The forward and inverse kinematics analysis and workspace calculation finally show that the mechanism meets the expected slurry spraying operation requirements and provides a new solution for the design of tunnel slurry spraying robot. The innovation of this paper lies in the mechatronic design of the whole equipment, which breaks the limitation of the existing similar equipment with joint structure only. And a new mechanical structure is proposed, which can realize the movement precision of the nozzle under the premise of guaranteeing the slurry spraying process, realizing the goal of adapting to the harsh underground environment and meeting the needs of the project.

For this type of robot, the spraying quality meets the engineering quality standard and shows the reliability of the structure itself. It can initially meet the needs of TBM construction operation and replace the traditional manual control. However, the structure still shows certain shortcomings, such as a large amount of shotcrete rebound, part of the concrete solidified on the surface of the machine and the joints, resulting in a decline in the precision of the machine. Therefore, it is planned to design a removable shell to protect the structure itself.

Although the design and application of the mechanical structure has been completed, it needs to be improved for different working conditions and severe working conditions. The next step is to integrate machine vision and digital twin technology to realize tunnel model construction, tunnel environment recognition, vehicle positioning and spraying quality perception, and to establish a series of path planning system to realize fully automated operation of the machine to meet the urgent needs of automation and intelligence of TBM construction and slurry spraying.

## Data Availability

All data generated or analysed during this study are included in this published article [and its supplementary information] files. If there are other data requirements, answers can be obtained from the corresponding author.
